# Digitally Barcoding *Mycobacterium tuberculosis* Reveals *In Vivo* Infection Dynamics in the Macaque Model of Tuberculosis

**DOI:** 10.1128/mBio.00312-17

**Published:** 2017-05-09

**Authors:** Constance J. Martin, Anthony M. Cadena, Vivian W. Leung, Philana Ling Lin, Pauline Maiello, Nathan Hicks, Michael R. Chase, JoAnne L. Flynn, Sarah M. Fortune

**Affiliations:** a Department of Immunology and Infectious Diseases, Harvard T. H. Chan School of Public Health, Boston, Massachusetts, USA; b Ragon Institute of Massachusetts General Hospital, Massachusetts Institute of Technology and Harvard, Cambridge, Massachusetts, USA; c Department of Microbiology and Molecular Genetics, University of Pittsburgh School of Medicine, Pittsburgh, Pennsylvania, USA; d Department of Pediatrics, Children’s Hospital of Pittsburgh, University of Pittsburgh Medical Center, Pittsburgh, Pennsylvania, USA; Washington University—St. Louis School of Medicine

**Keywords:** *Mycobacterium tuberculosis*, bacterial barcode, granuloma, infection mapping, lung infection, macaque

## Abstract

Infection with *Mycobacterium tuberculosis* causes a spectrum of outcomes; the majority of individuals contain but do not eliminate the infection, while a small subset present with primary active tuberculosis (TB) disease. This variability in infection outcomes is recapitulated at the granuloma level within each host, such that some sites of infection can be fully cleared while others progress. Understanding the spectrum of TB outcomes requires new tools to deconstruct the mechanisms underlying differences in granuloma fate. Here, we use novel genome-encoded barcodes to uniquely tag individual *M. tuberculosis* bacilli, enabling us to quantitatively track the trajectory of each infecting bacterium in a macaque model of TB. We also introduce a robust bioinformatics pipeline capable of identifying and counting barcode sequences within complex mixtures and at various read depths. By coupling this tagging strategy with serial positron emission tomography coregistered with computed tomography (PET/CT) imaging of lung pathology in macaques, we define a lesional map of *M. tuberculosis* infection dynamics. We find that there is no significant infection bottleneck, but there are significant constraints on productive bacterial trafficking out of primary granulomas. Our findings validate our barcoding approach and demonstrate its utility in probing lesion-specific biology and dissemination. This novel technology has the potential to greatly enhance our understanding of local dynamics in tuberculosis.

## IMPORTANCE

Classically, *M. tuberculosis* infection was thought to result in either latent infection or active disease. More recently, the field has recognized that there is a spectrum of *M. tuberculosis* infection clinical outcomes. Within a single host, this spectrum is recapitulated at the granuloma level, where there can simultaneously be lesional sterilization and poorly contained disease. To better understand the lesional biology of TB infection, we digitally barcoded *M. tuberculosis* to quantitatively track the fate of each infecting bacterium. By combining this technology with serial PET-CT imaging, we can dynamically track both bacterial populations and granuloma trajectories. We demonstrate that there is little constraint on the bacterial population at the time of infection. However, the granuloma imposes a strong bottleneck on dissemination, and the subset of granulomas at risk of dissemination can be distinguished by physical features.

## INTRODUCTION

Tuberculosis (TB) poses a threat to global health, responsible for more than 10 million new cases of active disease and nearly 2 million deaths in the last year (1). However, these recognized cases of TB disease reflect only a small fraction of *Mycobacterium tuberculosis* infections, most of which result in a spectrum of outcomes that are clinically silent and collectively referred to as latent TB infection (LTBI) (2–4). These different courses of infection are characterized by a range of bacterial burdens and pathologies and correlate with reactivation risk (5–8). Recently, the field has focused on early interactions between the host and bacterium as potential drivers of the variable outcomes of *M. tuberculosis* infection (9). However, new tools are needed to dissect the local biology of *M. tuberculosis* infection, especially in the nonhuman primate (NHP) model, whose strengths are that it recapitulates the variable course of human infection and produces individual granulomas with pathology very similar to that found in humans (5, 10, 11).

Dissection of lesion course in macaques has been transformed by the use of positron emission tomography coregistered with computed tomography (PET/CT) and an [^18^F]fluorodeoxyglucose radiotracer ([^18^F]FDG) and validated in humans (8, 12, 13). Using this approach, we previously demonstrated that formation of disseminated lesions early after infection, by which we mean the first 6 weeks postinfection, is associated with development of active disease, whereas limited early dissemination is associated with maintenance of clinically latent infection, suggesting that early dissemination is critical in determining host outcome (12). From these studies, it was unclear whether early dissemination was the result of a global defect in immunity or due to loss of control by a single lesion. We have also shown that granulomas follow unique trajectories even within individual macaques, as evidenced by differences in killing efficacy (10). Thus, we hypothesized that individual granulomas might similarly vary in their risk of dissemination.

We have also shown that most lesions harbor the progeny of a single bacterial founder. However, this study was limited to very early time points (~4 weeks postinfection) and so did not assess the dynamics of dissemination (10). Moreover, as the previous study only used a panel of 8 bacterial strains, it did not allow us to unambiguously resolve the subsequent fate of each bacterium following infection.

To address the lack of tools available to answer these critical biological questions, we developed a genome barcoding system, allowing us to track the fate of each infecting bacillus. The population dynamics of other pathogens have been tracked by assessing change in genetic composition of a population, leveraging either natural or artificially introduced genetic variation (14). However, these infection models are typically characterized by a relatively large infecting inoculum and wide bottlenecks that make tracking of individual bacteria or viruses in a population infeasible, and instead investigators have tracked changes in the distribution of diversity in the population to estimate bottleneck size. In contrast, TB is a paucibacillary infection, where inocula of <20 organisms successfully establish infection, allowing us to track infection dynamics by directly following the fate of each bacterium that establishes infection. To do this, we developed a complex library of digitally barcoded *M. tuberculosis* such that each infecting organism carries a unique and quantifiable sequence identifier. In parallel, we developed a robust Python algorithm to reliably discriminate bacterial barcodes. By combining this barcoding system with serial [^18^F]FDG PET/CT in a macaque model, we provide the first quantitative map of within-host bacterial population dynamics focusing on *M. tuberculosis* infection and dissemination.

## RESULTS

### Barcoded bacterial library generation.

**FIG 1:**
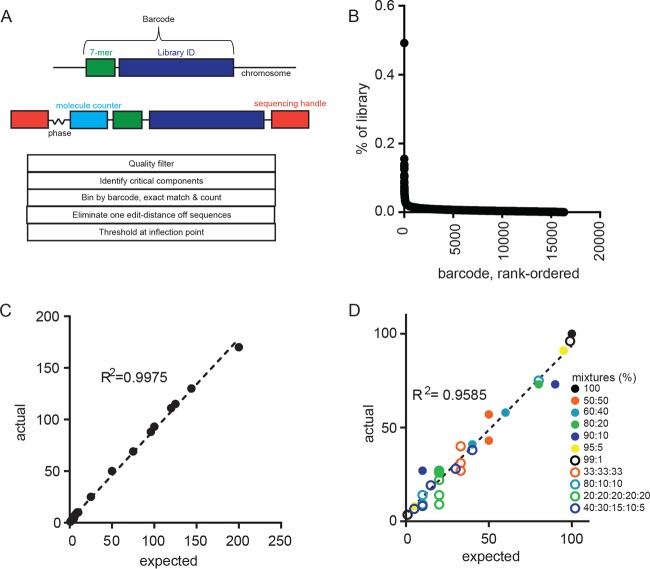
Barcode structure and pipeline validation. (A) Barcode structure in the *M. tuberculosis* genome (top) and after preparation for sequencing (middle). Schema of the BARTI pipeline for barcode identification, thresholding, and counting. (B) Abundance of individual barcodes in a single library. (C) Number of barcodes counted using BARTI from a known number of barcoded *Mycobacterium smegmatis* colonies at a read depth of >100,000. (D) Ratios of barcodes counted using BARTI (actual) from known mixtures of barcode-containing plasmids mixed at indicated ratios (expected) and sequenced at a read depth of >100,000. The dashed line indicates linear regression; *R*^2^ is the Spearman correlation.

We engineered a library of digitally barcoded plasmids that we introduced into *Mycobacterium smegmatis* and *M. tuberculosis* Erdman. The “barcode” consists of a random 7-mer and adjacent 75-mer library identifier tag stably inserted into the bacterial chromosome (15) (Fig. 1A). Using this approach, we were able to generate libraries of roughly 50,000 uniquely identifiable bacteria, ensuring a <2% chance that a barcode would be represented twice if 20 bacteria are randomly selected. To quantitate barcodes via sequencing, the barcode is amplified from isolated genomic DNA in two rounds of nested PCR steps. In the first round, a pool of four primers with a degenerate variable-length spacer anneals a random 9-mer “molecular counter” that allows us to enumerate PCR templates rather than amplicons (16). The degenerate variable-length spacer, or “phasing region,” introduces sequence variability necessary for Illumina-based sequencing of relatively low-complexity libraries (17). The second round of PCR completes the addition of sequencing adapters and multiplexing indices. Using this sequencing approach, we confirmed the diversity of the *M. tuberculosis* Erdman library and found that most barcodes were present at similar abundances (Fig. 1B). A few sequences appeared overrepresented; however, these represented only 0.5% of the total library and were not identified in any NHP used in this study.

Concurrently, we developed a custom Python pipeline, BARTI, to identify barcodes from Illumina sequencing data in complex biological samples (i.e., granulomas), where both sequences and the number of barcodes in a sample are unknown. The challenge in reading out randomized DNA barcodes is that errors inherent in sequencing create uncertainty in identifying unique barcode sequences. In previously published genomic barcoding approaches, this challenge was addressed by using only arrayed libraries of known barcodes, such that exact or near-exact sequence matching could be enforced (18). However, this approach significantly constrains the number of unique individuals represented in a population and precludes unambiguously tracking individual bacteria. In sequencing approaches where unknown barcodes are used, error correction methods have been developed to discriminate false sequences from true (19, 20), but the best approach to high-precision barcode counting is unclear.

To define the analytic path that provided the most accurate barcode quantitation, we arrayed a single colony of barcoded *M. smegmatis*, such that each colony contained only one barcode. We then generated and sequenced pools of colonies containing known numbers of barcodes. We expected that the number of barcode sequences identified should be equal to the number of colonies used in that sample’s preparation. Without error correction, as with other iterations of genome barcoding technology (18, 19), a much larger number of barcode sequences were identifiable in the sequencing data than were present in the input library. Strict read-quality filtering eliminated many, but not all, suspected erroneous sequences. Distance errors, wherein one or more bases in a barcode sequence are erroneously called, are common to these types of sequencing data. Often algorithms are applied to correct distance errors by condensing sequences that differ by one base from the dominant sequence in order to maintain the greatest read depth possible (21, 22). However, we found that condensing barcodes that differed by a single base in some cases skewed the estimation of barcode number. Thus, we chose to discard sequences that differed by one base from a more abundant barcode. This processed pool of barcode sequences still contained two populations of barcodes: abundant and putatively “true” and rare and putatively “false” barcodes representing persistent erroneous sequencing. By rank ordering the barcode sequences by template counts and taking the second derivative of the slope between each barcode to the next less abundant barcode, we found that the maximally negative point along this line described an inflection point that accurately separated the expected number of barcodes from sequencing artifacts. Using this analytic approach, we accurately quantitated the number of known bacterial barcodes in the pooled mixtures of arrayed mycobacterial colonies across a range of sequencing read depths (Fig. 1C; see Fig. S1 in the supplemental material). The sequencing pipeline also accurately quantitated both the number and relative abundance of barcode sequences present at unequal ratios when the least abundant barcodes were ≥1% of the population (Fig. 1D).

10.1128/mBio.00312-17.1 FIG S1  Barcode counting. (A) Pictorial example of BARTI thresholding of “real” barcodes from “noise” arising from sequencing artifacts. Unique barcodes from 25 picked *M. smegmatis* colonies are rank ordered by number of molecular counters. The inflection point can be visually described as the largest drop-off between two barcode sequences and is mathematically described and found in the Materials and Methods section. (B) BARTI has good accord between expected and actual found sequences across a range of read depths. Download FIG S1, PDF file, 0.2 MB. Copyright © 2017 Martin et al. 2017 Martin et al. This content is distributed under the terms of the Creative Commons Attribution 4.0 International license.

### Mapping *M. tuberculosis* infection dynamics in macaques.

**FIG 2:**
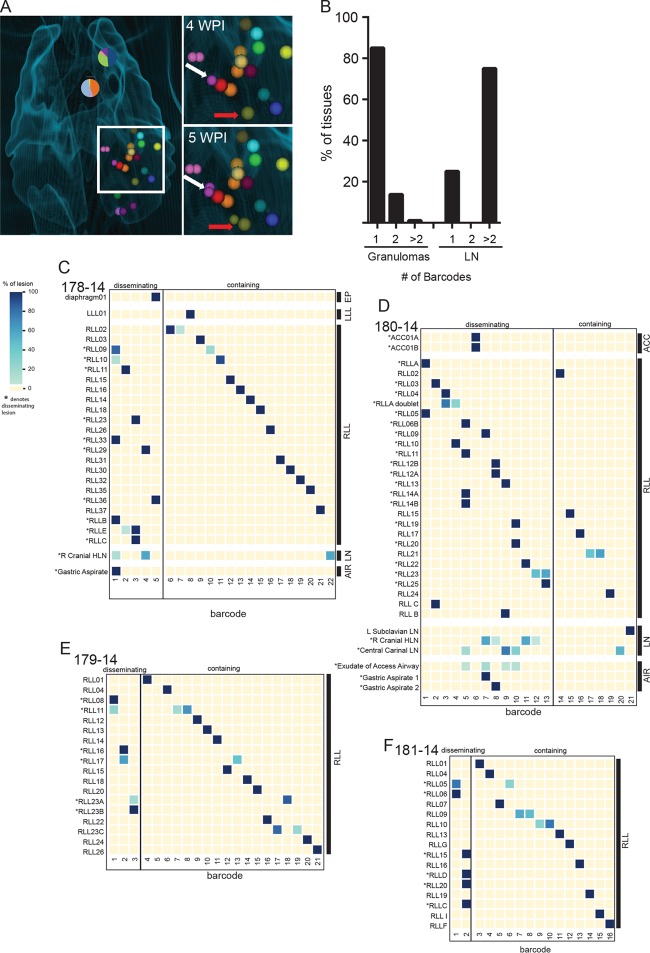
Contained and disseminated lesions from 15- to 20-week-infected macaques. (A) Thoracic CT scan from animal 180-14, with barcode composition in lymph nodes (pie charts) and granulomas (bubbles). The inset shows 4 and 5 weeks postinfection (p.i.) (WPI) with granulomas identifiable at those time points. The white arrow marks dissemination of the purple barcode from the founder lesion at 4 weeks p.i. to new lesion at 5 weeks p.i. The red arrow marks dissemination of the olive barcode from founder lesion at 4 weeks p.i. to a new lesion at 5 weeks p.i. (B) Quantification across all animals of number of unique barcode sequences found in granulomas and lymph nodes. (C to F) Contained and disseminated barcode sequences in all recovered CFU^+^ lesions arrayed according to a spatial distribution approximately from top to bottom of each lung lobe for the 4 macaques. (The macaque ID number is given in the top left of each graph.) Blue shading reflects the percentage of molecular counters out of the total for a lesion for that barcode. Numbered lesions were identified prenecropsy by scan, while lettered lesions were found during necropsy and often do not have corresponding *xyz* coordinates. Gastric aspirates in panel E were taken 13 days apart. Barcodes are arbitrarily numbered. RLL, right lower lobe; LLL, left lower lobe; ACC, accessory lung lobe; EP, extrapulmonary; LN, lymph node; AIR, airway.

To define bacterial population dynamics *in vivo*, we infected 4 macaques (Table 1) with a goal inoculum of ~20 CFU of barcoded Erdman, where the delivered inoculum as determined by plating was 11 ± 5 CFU, recognizing there is uncertainty in this number created by plating and counting very low numbers of a very dilute sample. We tracked temporal and spatial granuloma formation *in vivo* with serial [^18^F]FDG PET/CT for 15 to 20 weeks. Lesions were identified, labeled, and characterized for size and PET avidity (SUV) (12). Using the final prenecropsy PET/CT as a lesion map, we excised each of the lesions, plated their homogenates, and scraped and sequenced the resulting colonies to decode bacterial founder identity. By overlaying serial PET/CT scans with postsequencing barcode identity, we were able to create a systematic history of productive (i.e., culture-positive) lesion formation and dissemination in each animal (Fig. 2A).

**TABLE 1:** Parameters of barcoded *M. tuberculosis* Erdman infection and disease in macaques

Animal ID	No. of recovered barcodes	4-wk granuloma count (PET/CT)	Time to necropsy (wk)	Gross pathology score	Total CFU	Timing of PET/CT scans (wk)
178-14	22	26	19	21	23,979	3, 4, 6, 9, 12, 14, 18, 19
179-14	21	27	19	24	24,489	3, 4, 6, 8, 9, 12, 14, 18, 19
180-14	21	26	15	23	53,710	4, 5, 6, 10, 13, 15, 16
181-14	16	12	16	14	14,060	4, 5, 7, 10, 13, 16

### Concordance between estimated dose and number of bacteria that successfully establish infection.

In other pathogens, including *Salmonella* and HIV, barcode tracking has demonstrated that only a tiny fraction of the delivered organisms successfully establish infection (23, 24). *M. tuberculosis* is thought to be a more efficient pathogen, but the infective dose of *M. tuberculosis* has been difficult to define. We compared the number of unique barcodes identified in that animal with the target dose and estimated dose (by plating) of the inoculum to estimate the fraction of the inoculum that successfully established infection. The number of bacterial barcodes recovered from the animals closely matched the targeted inoculum (Table 1). Indeed, these counts were somewhat higher than the plated inoculum, variation we attribute to technical limitations in handling and plating very small numbers of organisms in a very dilute culture. Of note, all barcode sequences identified within a given animal differed at more than two bases, which given the bacterial mutation rate *in vivo* (25) would occur by mutation of the barcode so infrequently as to be irrelevant here (<10^−10^ bacteria/day). These data suggest that the early events of infection impose little to no bottleneck on the bacterial population and are consistent with the model that the infective dose of *M. tuberculosis* can be as low as one bacterium.

### A subset of granulomas disseminate to form new lesions.

We then sought to define bacterial population dynamics through the first 3 months of infection, encompassing the window of dissemination that we previously identified between 3 and 8 weeks of infection. We have previously shown that at 3 weeks postinfection, most granulomas harbor the progeny of a single founder (10). Here, we find that the majority of granulomas even at later time points contain the progeny of a single bacterial founder, as most lesions (85%) had only one barcode sequence (Fig. 2B). Thus, we find little evidence of interlesional mixing of the bacterial population even after a period of dissemination. Thoracic lymph nodes often contained more than one barcode (75%), as expected for sites draining the lung. However, only a minority of barcodes present in the lung were culturable from the lymph nodes, suggesting that not all granulomas productively disseminate to lymph nodes, at least at these later time points (Fig. 2C to F).

Barcode tracking defines two populations of granulomas: lesions containing bacteria that are derived from the same founder, which we interpret as the product of productive dissemination, and lesions harboring unique bacterial barcodes, which were “contained” (i.e., did not appear to have disseminated to form new culture-positive lesions). The patterns of dissemination varied across the four animals (Fig. 2C to F). In 3 macaques, the majority of lesions were contained. In one macaque (identification [ID] no. 180-14), ~80% of lesions were disseminated. This appeared to reflect failure of containment at multiple sites, as opposed to widespread dissemination from a single initial lesion. On the whole, however, this analysis demonstrates that while nearly all bacteria in an infecting inoculum successfully establish a culture-positive lesion, the majority do not contribute to further spread of infection. Across all 4 animals, only a small fraction (8.75%) of granulomas were able to seed multiple (≥3) new granulomas in lung and lymph nodes (Fig. 2C to F).

### Dissemination occurs primarily through local spread.

**FIG 3:**
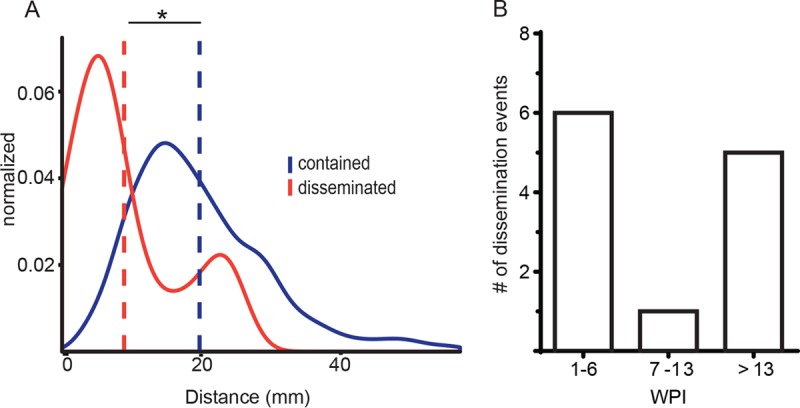
Spatial and temporal characteristics of dissemination. (A) Density histogram of the Euclidean distance of each disseminated lesion (red) to contained (blue) lesions with the same barcode sequence across all animals. The blue line is the Euclidean distance of all contained lesions to other lesions that do not contain the same barcode sequence. Data are only reflective of lesions for which *xyz* coordinates are known. The dashed line is the mean for each distribution, *; *P* < 0.05 by Welch’s *t* test. (B) Number of lung disseminating events across all animals at the indicated time points (weeks postinfection [WPI]). The timing reflects definitive dissemination events matched with barcode and serial imaging.

We sought to better understand these patterns of dissemination by defining the spatial dynamics of bacterial spread. Using 3-dimensional lesional coordinates from prenecropsy PET/CT scans, we interrogated the spatial relationships between disseminated lesions containing the same barcode compared to the population of contained lesions (see Fig. S2 in the supplemental material). Lesions that share barcodes are closer together than the population of contained lesions (distance between mean contained, 19.657 mm; distance between mean disseminated, 8.87 mm) and are significantly different populations (*D* = 0.615, *P* = 2.82e−9) (Fig. 3A). Thus, most dissemination appears to result from local spread.

10.1128/mBio.00312-17.2 FIG S2  Distance between lesions. Shown is the Euclidean distance between each lung granuloma and every other lesion in that animal. Contained lesions are in blue and disseminated lesions in red. Download FIG S2, PDF file, 0.6 MB. Copyright © 2017 Martin et al. 2017 Martin et al. This content is distributed under the terms of the Creative Commons Attribution 4.0 International license.

Matching timing data from serial scans with barcode identity, we also established the temporal dynamics of dissemination. Consistent with previous findings (12), there is substantial dissemination within the first 6 weeks of infection. After this window of early dissemination, there is a period of apparent quiescence and then a second wave of granuloma spread (Fig. 3B).

### Disseminated lesions are larger than contained lesions.

The variability in the patterns of dissemination was striking as even the animal allowing extensive dissemination was not identifiable by clinical course. These animals were also not distinguished by their total bacterial burdens at necropsy (Table 1), which were similar to the average total CFU compared to our historical data (*n =* 18 cynomolgus macaques [7, 12]; range, 0 to 222,864; mean, 34,338; median, 6,613).

These data suggested that dissemination is driven by lesional as well as global features. We therefore sought to identify lesional characteristics that distinguish lesions containing bacteria from those disseminating them. We compared the populations of T cells between these sets of lesions but found only minor differences in the frequency of cytokines produced by T cells following *ex vivo* stimulation (see Fig. S3 in the supplemental material). However, as dissemination and necropsy are separated by weeks to months, the quality of the immune response likely changed dramatically. Our previous data support that both bacterial burden and immune responses in granulomas evolve over time (5, 10, 11). Since granulomas can only be obtained at necropsy for assessment of bacterial and immune characteristics, we cannot determine in this study how local T cell responses at the time of dissemination affect the fate of the lesion.

10.1128/mBio.00312-17.3 FIG S3  Frequency of cytokine-producing T cells for IFN-γ (A), IL-2 (B), IL-6 (C), IL-10 (D), IL-17A (F), and TNF-α show minor differences between contained and disseminated lesions. Each symbol is a granuloma (Gran). Analyzed contained (*n =* 7) and disseminated (*n =* 12) lesions had a minimum of 30 CD3^+^ T cells after processing and gating in FlowJo version 9.9.5 for analysis (*, *P* =0.0.283; **, *P* = 0.0098). Statistics for panels A to F were determined by Mann-Whitney test. Download FIG S3, PDF file, 0.9 MB. Copyright © 2017 Martin et al. 2017 Martin et al. This content is distributed under the terms of the Creative Commons Attribution 4.0 International license.

**FIG 4:**
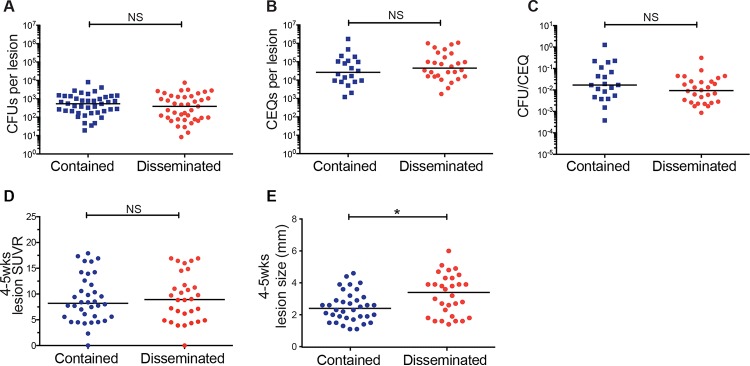
Granuloma size early during the early phase differentiates dissemination. (A) Bacterial burden (CFU) from granulomas obtained at necropsy (15 to 20 weeks p.i.) of contained (*n =* 41) and disseminated (*n =* 42) lesions from four barcoded macaques. Data are reflective of lesions initiating dissemination as determined by temporal PET/CT analysis. In instances where the precise founder is not known, all involved lesions were classified as disseminated. (B) Total bacterial load (live plus dead [CEQ]) of contained (*n =* 20) and disseminating (*n =* 28) lesions. (C) Bacterial killing determined by the CFU/CEQ ratio for contained (*n =* 20) and disseminating (*n =* 28) lesions. (D) Granuloma FDG avidity (SUVR) and (E) size (millimeter) of contained (*n =* 36) and disseminating (*n =* 30) lesions at 4 to 5 weeks postinfection as assessed by PET/CT (*, *P* = 0.0154). In panels A to E, each symbol represents a granuloma. Statistics for panels A to E were determined by the Mann-Whitney test.

PET/CT imaging allows us to interrogate lesional biology over the course of infection, not simply at the time of necropsy. To begin to identify lesional features that correlate with fate, we screened disseminated and contained granulomas for differences in bacterial burden (Fig. 4A), cumulative bacterial load (chromosomal equivalent quantification [CEQ]) (Fig. 4B), and bacterial killing (CFU/CEQ) (Fig. 4C) at the time of necropsy. None of these parameters, determined by necessity at 15 to 20 weeks postinfection, predicted the risk of dissemination. Next, we evaluated [^18^F]FDG PET/CT characteristics, including FDG avidity (SUVR) (Fig. 4D) and size (Fig. 4E) for each granuloma over the course of infection. While SUV, a measure of metabolic uptake of [^18^F]FDG and a proxy for inflammation, did not reveal any differences between the two lesion fates, granuloma size as measured by PET/CT early in infection (at 4 to 5 weeks) differentiated the two fates (*P* = 0.0154); larger lesions early in infection were associated with a higher risk of dissemination and formation of new culture-positive lesions. We assessed whether the size of the granuloma at 4 to 5 weeks was correlated with CFU at the time of necropsy (15 to 19 weeks) and found only a weak correlation (*r*^2^ = 0.145, *P* = 0.001) (see Fig. S4A in the supplemental material). To determine whether size and CFU were associated at the earlier time point, we analyzed data from a separate set of 6 macaques (not infected with barcoded strains) and found an even weaker correlation between size at 4 to 5 weeks postinfection and CFU at 5 to 6 weeks postinfection (*r*^2^ = 0.066, *P* = 0.039) (Fig. S4B). Thus, there is little correlation between size and bacterial burden of granulomas at early time points.

10.1128/mBio.00312-17.4 FIG S4  Size is not associated with granuloma bacterial burden. There is a very weak correlation between granuloma size, as measured by PET/CT at 4 to 5 weeks postinfection, and CFU in granulomas necropsied at both 15 to 19 weeks postinfection (A) (*r*^2^ = 0.140, *P* = 0.001, *n =* 71) and 5 to 6 weeks postinfection (B) (*r*^2^ = 0.066, *P* = 0.0391). Linear regressions were performed in Prism 6. Download FIG S4, PDF file, 0.7 MB. Copyright © 2017 Martin et al. 2017 Martin et al. This content is distributed under the terms of the Creative Commons Attribution 4.0 International license.

## DISCUSSION

To resolve the within-host bacterial population dynamics that contribute to the spectrum of *M. tuberculosis* infection, we generated a novel library of digitally barcoded *M. tuberculosis* and an associated Python pipeline for their identification and tracking in biological samples from macaques. This barcode library and accompanying pipeline overcome significant hurdles inherent in this type of analysis. By pairing information from serial PET/CT scanning with the sequence identities of bacteria from infected tissue, we have an unparalleled level of resolution of within-host infection dynamics at a lesional level. Genome tagging has been used successfully to ask many questions about the population behavior of bacterial and viral infections, but to date, all of these strategies involved tracking of pools of known barcodes via exact sequence matching to distinguish barcodes. We have developed an approach to accurately identify and quantitate individuals pulled from a highly complex library in which the sequence identifiers are not known *a priori*. These tools allow us to confidently track the descendants of individual bacteria and the population as a whole.

In this study, we sought to define constraints on the bacterial population from inoculation through the first 5 months postinfection. Importantly, we found no evidence of significant impediments to the establishment of *M. tuberculosis* infection, in that the number of individual bacteria identified by digital barcode and the number of initial granulomas observed by PET/CT are similar to the estimated infectious dose. While this study cannot assess all constraints on the bacterial population associated with transmission—especially those associated with aerosolization—the findings are consistent with the hypothesis that the natural infective dose of *M. tuberculosis* could be as low as a single organism (26, 27). These data highlight the limited capacity of the earliest innate responses to prevent establishment of infection.

Although the innate response does not appear to limit initial infection, it does appear to constrain early dissemination. Indeed, we found evidence of significant constraints on dissemination, indicating that there is a bottleneck imposed on the bacterial population at the point of escape from the granuloma. In most animals, only a minority of granulomas seed a second, culture-positive granuloma. Previous work in the zebrafish model found that in contrast, dissemination was the rule and not the exception (28).

Our data suggest additional facets to the lesional variability that we have previously documented in terms of bactericidal capacity (10). We found that there is variability in the extent of dissemination that is an independent marker of disease course, distinct from total bacterial, clinical, or radiographic measures of disease. We demonstrated that granuloma size assessed by [^18^F]FDG PET/CT in the first few weeks postinfection correlates with risk of dissemination. Although a simple explanation for the relationship of size with dissemination risk would be bacterial burden, our data do not support this at early time points. Instead, we speculate that size is an independent biological feature and that physical expansion increases the bacterium’s access to avenues of spread. Notably, we previously observed that at 6 weeks postinfection, lesion size was greater in macaques that would subsequently develop active TB (9), and here we report it as a correlate for dissemination reiterating the importance of the events during the first 6 weeks of infection on outcome (12). Finally, most instances of productive dissemination were to sites less than 10 mm away, indicating that spread of *M. tuberculosis* is often local.

Interestingly, spread to lymph nodes is also highly variable across infected macaques (10, 11, 29). Only 15% of barcodes were represented in the lymph nodes that were culture positive. (We recovered data from only 2 macaques in this study.) One current limitation of this work is that we are only able to confidently assess and sequence barcodes from live bacteria present at necropsy—i.e., those that grow on plates and not directly from tissue homogenate. This is in part due to the paucity of bacterial genomic material in these samples, which makes the distinction between true and erroneous barcodes in the amplified library challenging. Clearly, productive infection of the lymph node by every bacterial population in the animal is not required for the lymph node to sample and present the antigens from these populations. However, these data do raise questions about the extent of compartmentalization during *M. tuberculosis* infection and how that might influence generation of adaptive immune responses.

Questions like those of antigenic compartmentalization are only important if there are important functional differences between the bacteria in different sites within the host. Our work to date has demonstrated that lesional bacterial populations are isolated from the earliest points in infection. Our previous studies of bacterial mutability (25) have demonstrated that the rates of genetic mutation are too low to generate sufficient variation to account for observed differences in lesional course at these early time points during infection—although they do not preclude genetic differentiation over longer time periods. However, we cannot rule out the possibility of distinct patterns of transcriptional adaptation or epigenetic inheritance in these isolated bacterial populations that will be the subject of future studies. One important lesson from these studies is the fact that the barcodes recovered from gastric and bronchoalveolar lavage samples, which are taken as proxies for the sputum in the NHP model, represent only a fraction of the bacterial barcodes (3.75%) present in the animal (Fig. 2C and D). These data are consistent with clinical studies demonstrating that organisms with different drug resistance patterns can be isolated from the sputum of individual patients and highlight the need to be cautious in interpreting the sputum bacteria as representative of the entire bacterial population within an individual (30, 31).

Previously we reported heterogeneity of granuloma features and bacterial control within a single infected animal, and here we reveal another layer of heterogeneity and complexity between different granulomas. Our findings reveal novel avenues of research to probe both host and bacterial factors that influence such disparate granuloma fates. These new tools will allow further characterization of the dynamic local interactions of bacteria and host that ultimately govern both lesion fate and patient outcome. Furthermore, the use of genome barcoding and tagging technologies has broad application, from development to cancer, and the new tools presented here will provide solutions to digitally tracking cells in a variety of fields.

## MATERIALS AND METHODS

### Barcode generation.

Primers (CM29/CM30; see the primers listed in Table S1 in the supplemental material) using a string of degenerate bases were used to amplify qTags (15) from parent vectors and were restriction digested and cloned into pJeb402 with KpnI and XbaI. Each library (single qTag) was constructed in *Escherichia coli* and plated at 4× coverage before plates were scraped, maxi-prepped (Qiagen), and transformed into *M. tuberculosis* Erdman or *M. smegmatis* mc^155^ at 4× coverage. Libraries were then scraped, passed through a 5-µm-pore filter (Millex), sonicated for single-cell suspension, and mixed at equal optical densities at 600 nm (OD_600_) to generate infectious libraries containing a pool of three library ID numbers.

10.1128/mBio.00312-17.5 TABLE S1  Primer sequences for *M. tuberculosis* barcode generation and Illumina sequencing. A list of the primer sequences used for barcode cloning and Illumina sequencing is shown. Download TABLE S1, PDF file, 0.3 MB. Copyright © 2017 Martin et al. 2017 Martin et al. This content is distributed under the terms of the Creative Commons Attribution 4.0 International license.

### Sequencing.

Bead-beaten and phenol-chloroform-purified genomic DNA was diluted to 10 ng/µl and amplified using Phusion polymerase through two rounds of PCR of 12 cycles each. Primers are indicated in Table S1. Fragments of the appropriate size were gel purified, quantified using the Clonetech NGS kit, and sequenced on an Illumina MiSeq using V2 chemistry.

### Macaque infections, PET/CT imaging, and tissue excision.

Four adult cynomolgus macaques (*Macacca fasicularis*) were obtained from Valley Biosystems (Sacramento, CA) and screened for *M. tuberculosis* and other comorbidities during a month-long quarantine. Each macaque had a baseline blood count and chemical profile and was housed according to the standards listed in the Animal Welfare Act and the *Guide for the Care and Use of Laboratory Animals*. All animals were infected with barcoded *M. tuberculosis* strain Erdman via bronchoscopic instillation as previously published (5, 32) and received an inoculum of 11 ± 5 CFU (determined by plating a direct sample of the inoculum and counting CFU after 3 weeks) (Table 1). All animals were followed with serial 2-deoxy-2-[^18^F]fluoro-d-glucose ([^18^F]FDG) PET/CT imaging as previously described (10, 12, 13) to identify and track lesion formation and progression over time. As before, lesions were individually characterized by their date of establishment (scan date), size (millimeters), and relative metabolic activity as a proxy for inflammation ([^18^F]FDG standard uptake normalized to muscle [SUVR]). Lesions of ≥1 mm can be discerned by our PET/CT equipment. At necropsy, the final PET/CT scan was used to identify all lesions, and careful dissection of each lung lobe, all thoracic and peripheral lymph nodes, spleen, liver, and kidney was performed. All PET/CT-identified lesions and any additional granulomas (e.g., <1 mm) were harvested for analysis. To avoid barcode cross-contamination, individual granulomas were separately excised and processed. Gross pathology scoring was performed as previously described (5) to obtain an overall disease score for each monkey.

### Isolation and preparation of bacteria from tissue samples.

Following removal at necropsy, each lesion was homogenized and plated for bacterial burden on 7H11 agar supplemented with oleic albumin dextrose catalase (OADC). A small portion of homogenate was frozen for chromosomal equivalent quantification (CEQ) analysis (below). After 3 weeks of incubation, the plates were counted for CFU, and colonies were pooled and scraped into 7H9 supplemented with OADC and 20% Tween 80. Each plate was scraped into a separate tube and frozen in 5 ml of 7H9 at −80°C to await genomic DNA extraction and sequencing.

### Extraction of *M. tuberculosis* genomes and CEQ.

*M. tuberculosis* genomic extraction and CEQ were performed as previously published (7, 10). Briefly, gDNA was extracted with phenol-chloroform-isoamyl alcohol (25:24:1 [Invitrogen]) with an intermediate bead-beating step using 0.1-mm-diameter zirconia-silica beads (BioSpec Products, Inc.). CEQ was assessed relative to a serially diluted standard curve of *M. tuberculosis* genomic DNA using quantitative real-time PCR performed in triplicate on an iQ5 Multicolor real-time PCR detection system (Bio-Rad Laboratories, Inc.) and a 384-well 7900HT fast real-time PCR system (Applied Biosystems). Quantification of CEQ used the previously published primer-probe mixture and TaqMan Universal master mix II (Thermo, Fisher Scientific). Acceptable real-time PCR efficiency for each run was kept between 90% and 110%.

### Bioinformatics analysis.

Data were analyzed using custom Python pipeline available on GitHub (https://github.com/sarahfortunelab/barcodetracking). The pipeline iterates through the raw FASTQ files generated from Illumina sequencing for each indexed sample. We first identify library ID, barcode, and molecular counter features using constant “handle” sequences as search motifs. We then filtered the data to obtain high-quality reads that (i) have all features present and (ii) have a maximum probability of base-calling error of 0.001 (equivalent to a minimum base quality score of least 30 or Phred score of 63). As an additional check, we grouped reads that passed quality control and counted molecular counter copies to check the extent of skewing. For each sample, we also tallied the number of unique molecular counters for each sequence combination and normalized counts to the total molecular counts in the sample. Using the aforementioned percentage counts as a metric for library-ID-barcode abundance, we computed the true-false-determining threshold in the pipeline using a recursive approach. In each round, we first computed and sorted the relative abundance in descending order. Second, we iterated through the barcodes that passed the previous threshold and for each eliminated less-abundant variants that differed by one position from the former. Third, we calculated the threshold dividing true and false barcodes by using a modified concavity approach. Once the threshold reached a stable point, we applied the final threshold to return the set of true barcodes.

### T cell flow cytometry and intracellular cytokine staining.

At necropsy, granulomas were processed into single-cell suspensions with sterile PBS. Portions of these suspensions were used for T cell profiling and cytokine analysis. Tissue suspensions were stimulated with *M. tuberculosis* CFP10 and ESAT 6 (BEI Resources, Manassas, VA) and brefeldin A (GolgiPlug [BD Biosciences]) in RPMI (Lonza, Walkersville, MD) supplemented with 1% l-glutamine and 1% HEPES (Sigma, St. Louis, MO) for 3.5 h. The final concentration of *M. tuberculosis*-specific peptides was 2.5 μg/ml. Cells were first stained with LIVE/DEAD fixable blue dead cell staining kit (Thermo, Fisher Scientific) for viability and then stained with the T cell-specific marker CD3, using the anti-human CD3 (clone SP34-2 [BD Biosciences]) antibody in a standard fluorescence-activated cell sorter (FACS) buffer. Following surface staining described above, the cells were fixed and permeabilized (Cytofix/Cytoperm [BD]) and washed with BD Perm/Wash buffer (BD Biosciences). Incubations were performed according to the manufacturer’s recommendations. Cells were then stained with the following intracellular cytokine stains: anti-human gamma interferon (IFN-γ) (clone B27 [BD Biosciences]), anti-human tumor necrosis factor alpha (TNF-α) (clone Mab11 [eBiosciences]), anti-human interleukin-2 (IL-2) (clone MQ1-17H12 [BD Biosciences]) and IL-6 (clone MQ2-6A3 [BD Biosciences]), anti-human IL-10 (clone JES3-9D7 [eBiosciences]), and anti-human IL-17A (clone eBio64CAP17 [eBiosciences]). Flow cytometry was performed on an LSR II (BD) and analyzed using FlowJo software version 9.9.5 (Treestar, Inc., Ashland, OR). Size (forward scatter [FSC]) and granularity (side scatter [SSC]) were used to isolate the lymphocyte population during the cytometry. All cytokine data presented were gated on CD3^+^ T cells.

### Statistical analysis.

Statistical analysis was performed in GraphPad Prism, JMP, and R. Significance was found when *P* was <0.05.
