# Supplementary figures and images for: Digitally Barcoding Mycobacterium tuberculosis Reveals In Vivo Infection Dynamics in the Macaque Model of Tuberculosis
Source: mBio. 2017 May 9;8(3):e00312-17. doi: 10.1128/mBio.00312-17 (PMC5424202; doi:10.1128/mBio.00312-17)

Supplementary Figure 1

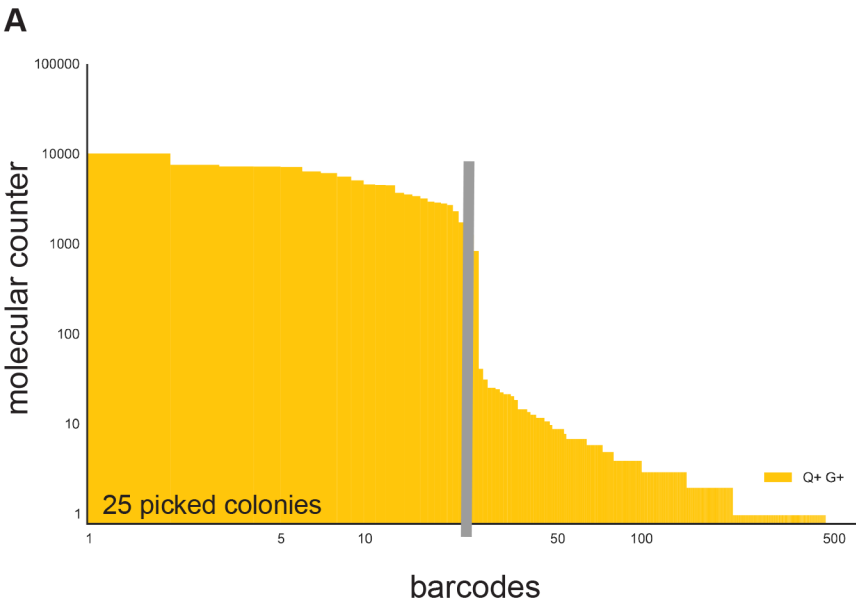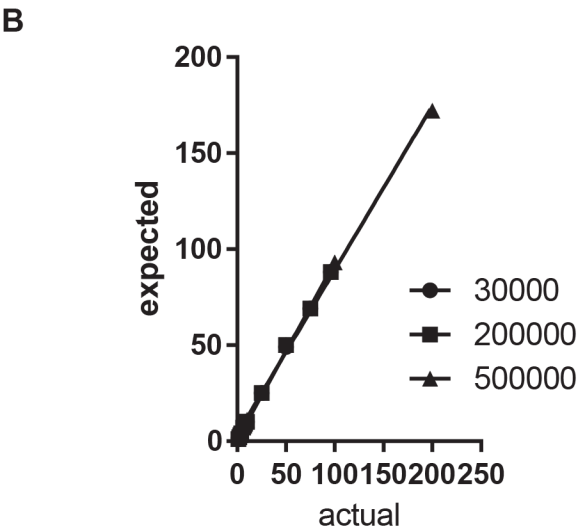

|            |        |        |        |
|------------|--------|--------|--------|
| read depth | 30000  | 200000 | 500000 |
| R square   | 0.9986 | 0.9981 | 0.9975 |

Supplement: FIG S1 [file mbo002173302sf1.pdf]

Supplementary Figure 2

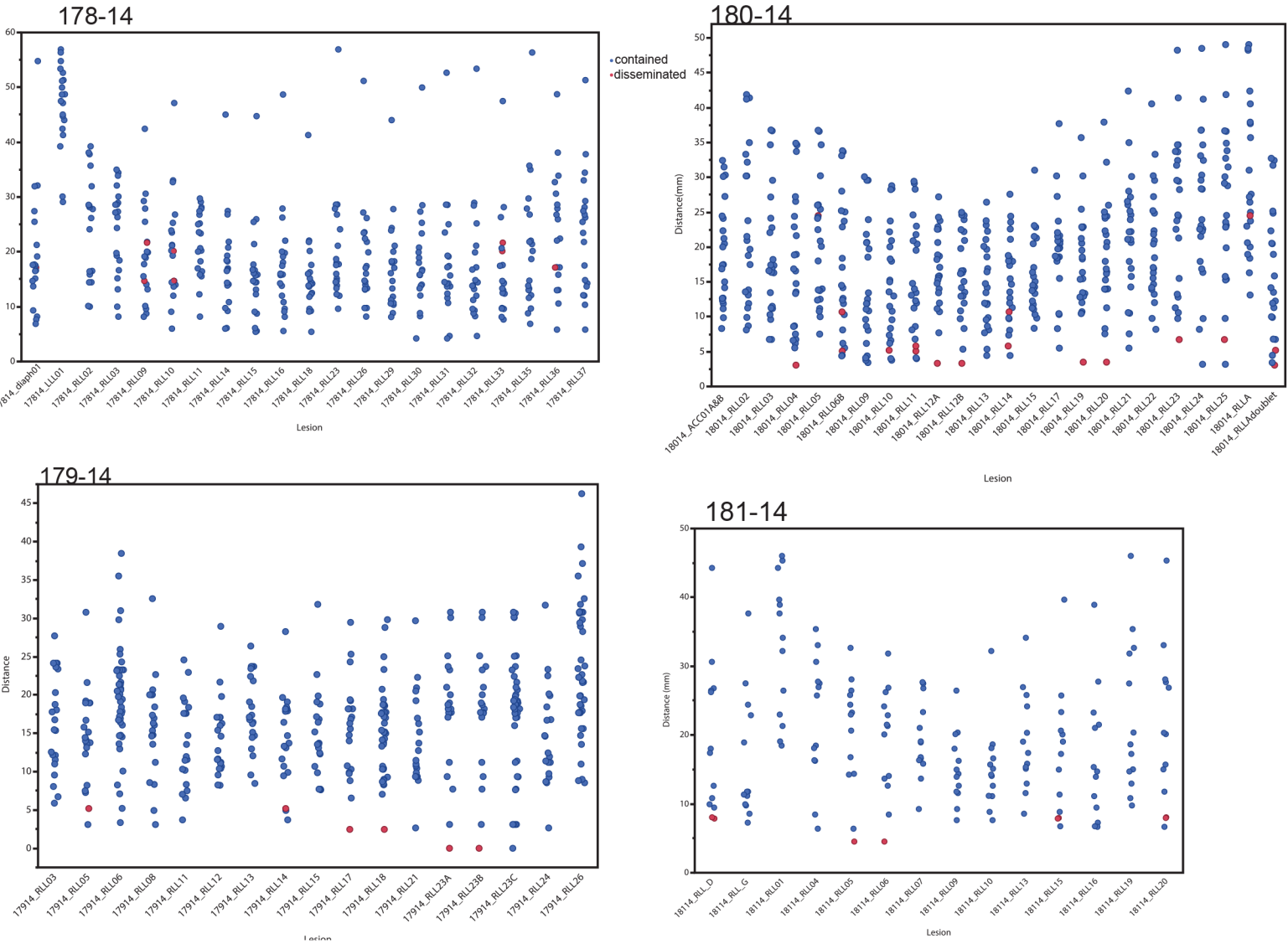

Supplement: FIG S2 [file mbo002173302sf2.pdf]

Supplementary Figure 3

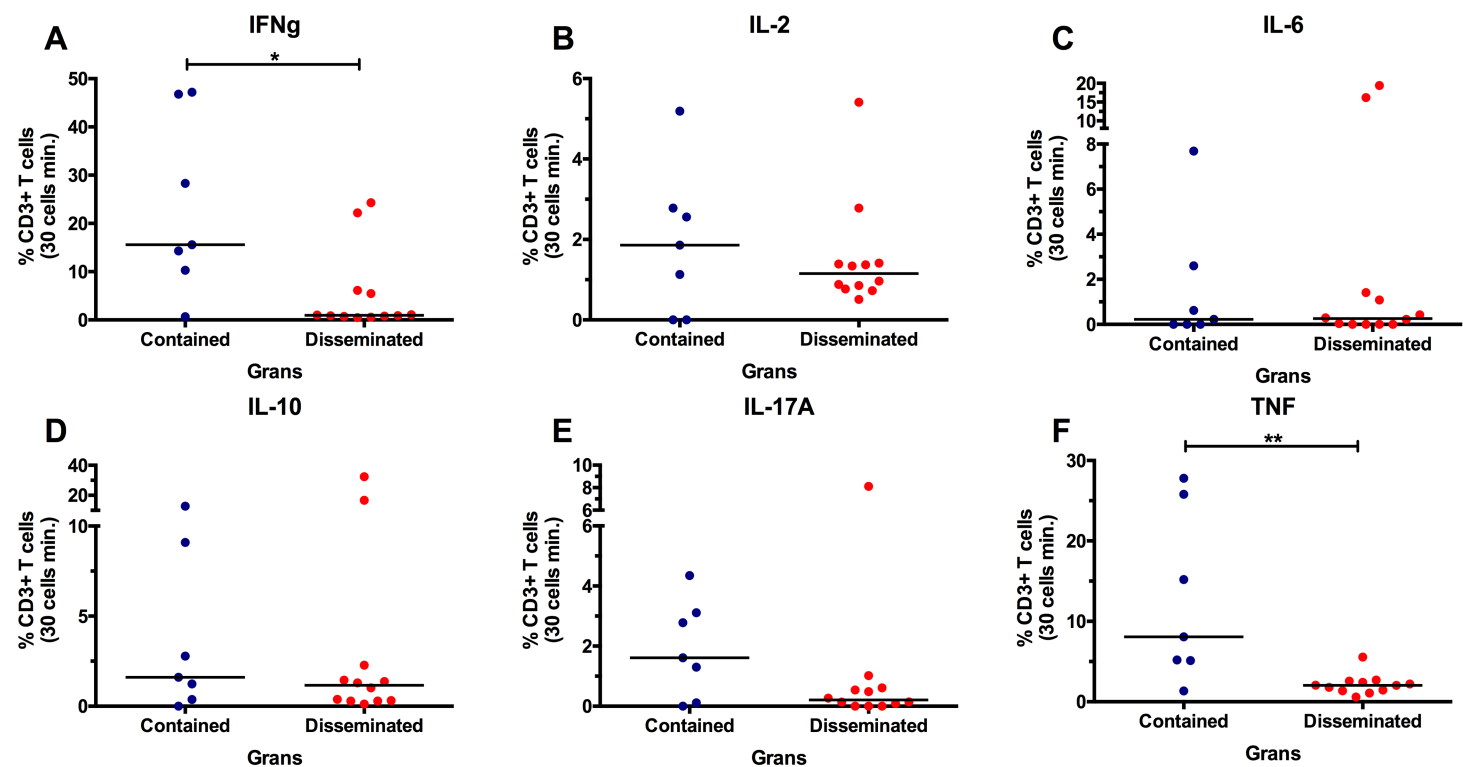

Supplement: FIG S3 [file mbo002173302sf3.pdf]

Supplementary Figure 4

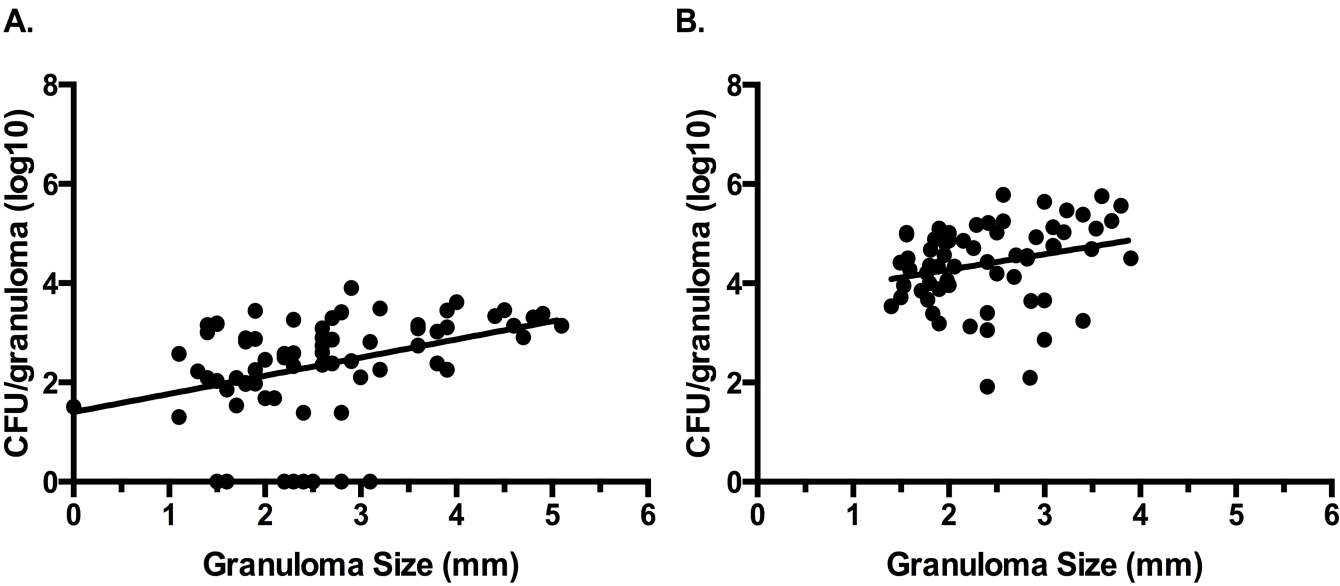

Supplement: FIG S4 [file mbo002173302sf4.pdf]
