# Supplementary material for: Digitally Barcoding Mycobacterium tuberculosis Reveals In Vivo Infection Dynamics in the Macaque Model of Tuberculosis
Source: mBio. 2017 May 9;8(3):e00312-17. doi: 10.1128/mBio.00312-17 (PMC5424202; doi:10.1128/mBio.00312-17)
Supplement: TABLE S1 [file mbo002173302st1.pdf]

Supplementary Table 1

Name	*Sequence*
**Barcode Generation Primers**	
CM29	GTACGAGGTACCCGANNNCNNNNaattcgatggcctagctgg
CM30	GGCCTATCTAGAgaccacaacggtttccatatg
**Illumina Library Primers**	
step 1	
Ftotal1	CCCTACACGACGCTCTTCCGATCTNCNNNCNNNCNNN
Ftotal2	CCCTACACGACGCTCTTCCGATCTNNCNNNCNNNCNNN
Ftotal3	CCCTACACGACGCTCTTCCGATCTNNNCNNNCNNNCNNN
Ftotal4	CCCTACACGACGCTCTTCCGATCTCNNNCNNNCNNN
Rtotal1	GTGACTGGAGTTCAGACGTGTGCTCTTCCGATCNGagaccacaacggtttccatatg
Rtotal2	GTGACTGGAGTTCAGACGTGTGCTCTTCCGATCNNNGAgaccacaacggtttccatatg
Rtotal3	GTGACTGGAGTTCAGACGTGTGCTCTTCCGATCNNNNGAgaccacaacggtttccatatg
Rtotal4	GTGACTGGAGTTCAGACGTGTGCTCTTCCGATCGAgaccacaacggtttccatatg
step 2	
F501	AATGATACGGCGACCACCGAGATCTACACTAT AGCCTACACTCTTTCCCTACACGACGCTCTTCC
F502	AATGATACGGCGACCACCGAGATCTACACATA GAGGCACACTCTTTCCCTACACGACGCTCTTCC
F503	AATGATACGGCGACCACCGAGATCTACACCCT ATCCTACACTCTTTCCCTACACGACGCTCTTCC
F504	AATGATACGGCGACCACCGAGATCTACACGGC TCTGAACACTCTTTCCCTACACGACGCTCTTCC
F505	AATGATACGGCGACCACCGAGATCTACACAGG CGAAGACACTCTTTCCCTACACGACGCTCTTCC
F506	AATGATACGGCGACCACCGAGATCTACACTAATCTTAACACTCTTTCCCTACACGACGCTCTTCC
F507	AATGATACGGCGACCACCGAGATCTACACCAGGACGTACACTCTTTCCCTACACGACGCTCTTCC
F508	AATGATACGGCGACCACCGAGATCTACACGTACTGACACACTCTTTCCCTACACGACGCTCTTCC
R701	CAAGCAGAAGACGGCATACGAGATCGAGTAATGTGACTGGAGTTCAGACGTGTGC
R702	CAAGCAGAAGACGGCATACGAGATTCTCCGGAGTGACTGGAGTTCAGACGTGTGC
R703	CAAGCAGAAGACGGCATACGAGATAATGAGCGGTGACTGGAGTTCAGACGTGTGC
R704	CAAGCAGAAGACGGCATACGAGATGGAATCTCGTGACTGGAGTTCAGACGTGTGC
R705	CAAGCAGAAGACGGCATACGAGATTTCTGAATGTGACTGGAGTTCAGACGTGTGC
R706	CAAGCAGAAGACGGCATACGAGATACGAATTCGTGACTGGAGTTCAGACGTGTGC
R707	CAAGCAGAAGACGGCATACGAGATAGCTTCAGGTGACTGGAGTTCAGACGTGTGC
R708	CAAGCAGAAGACGGCATACGAGATGCGCATTAGTGACTGGAGTTCAGACGTGTGC
R709	CAAGCAGAAGACGGCATACGAGATCATAGCCGGTGACTGGAGTTCAGACGTGTGC
R710	CAAGCAGAAGACGGCATACGAGATTTCGCGGAGTGACTGGAGTTCAGACGTGTGC
R711	CAAGCAGAAGACGGCATACGAGATGCGCGAGAGTGACTGGAGTTCAGACGTGTGC
R712	CAAGCAGAAGACGGCATACGAGATCTATCGCTGTGACTGGAGTTCAGACGTGTGC
